# Analysis of demographics and the impact of adjuvant radiotherapy on a nationwide cohort of patients with high-grade spinal meningiomas

**DOI:** 10.1093/noajnl/vdae018

**Published:** 2024-02-05

**Authors:** Victor Gabriel El-Hajj, Abdul Karim Ghaith, Ryan H Nguyen, Neil Nazar Al-Saidi, Harry Hoang, Stephen P Graepel, Adrian Elmi-Terander, Eric J Lehrer, Paul Brown, Mohamad Bydon

**Affiliations:** Department of Clinical Neuroscience, Karolinska Institutet, Stockholm, Sweden; Mayo Clinic Neuro-Informatics Laboratory, Mayo Clinic, Rochester, Minnesota, USA; Department of Neurological Surgery, Mayo Clinic, Rochester, Minnesota, USA; Mayo Clinic Neuro-Informatics Laboratory, Mayo Clinic, Rochester, Minnesota, USA; Department of Neurological Surgery, Mayo Clinic, Rochester, Minnesota, USA; Mayo Clinic Neuro-Informatics Laboratory, Mayo Clinic, Rochester, Minnesota, USA; Department of Neurological Surgery, Mayo Clinic, Rochester, Minnesota, USA; Mayo Clinic Neuro-Informatics Laboratory, Mayo Clinic, Rochester, Minnesota, USA; Department of Neurological Surgery, Mayo Clinic, Rochester, Minnesota, USA; Mayo Clinic Neuro-Informatics Laboratory, Mayo Clinic, Rochester, Minnesota, USA; Mayo Clinic Neuro-Informatics Laboratory, Mayo Clinic, Rochester, Minnesota, USA; Department of Neurological Surgery, Mayo Clinic, Rochester, Minnesota, USA; Department of Clinical Neuroscience, Karolinska Institutet, Stockholm, Sweden; Capio Spine Center Stockholm, Löwenströmska Hospital, Upplands-Väsby, Sweden; Department of Radiation Oncology, Mayo Clinic, Rochester, Minnesota, USA; Department of Radiation Oncology, Mayo Clinic, Rochester, Minnesota, USA; Mayo Clinic Neuro-Informatics Laboratory, Mayo Clinic, Rochester, Minnesota, USA; Department of Neurological Surgery, Mayo Clinic, Rochester, Minnesota, USA

**Keywords:** adjuvant radiotherapy, high grade, overall survival, spinal meningiomas, surgery

## Abstract

**Background:**

Although typically benign, 5% of spinal meningiomas (SMs) present with higher-grade features (World Health Organization grades 2 and 3). High-grade SMs are poorly studied and the role of adjuvant radiotherapy in their management remains controversial. We hence aimed to study the demographic characteristics of this rare tumor and investigate the outcomes associated with the use of surgery with adjuvant therapy in contrast to surgery alone.

**Methods:**

The National Cancer Database was queried for patients with SMs from 2004 to 2017. Basic statistics were used to identify differences between low- and high-grade tumors in terms of baseline characteristics. Surgery with and without adjuvant radiotherapy were compared after (1:1) propensity-score matching. Kaplan–Meier survival analysis was conducted to study overall survival. All analyses were performed on R.

**Results:**

A total of 13 184 patients diagnosed with SMs were included, of whom only 5% (*n* = 669) had high-grade SMs. Patients with high-grade SMs presented at a younger median age (57 years [IQR: 44–68] versus 65 years [54–75]; *P* < .001) and were more commonly males (33% vs 20%; *P* < .001). After propensity-score matching, survival analysis revealed similar overall survival outcomes in patients with high-grade SM undergoing both surgery and radiotherapy as compared to those only receiving surgery (*P* = .19).

**Conclusions:**

This study reveals major demographic differences between high- and low-grade SMs. There were no benefits associated with the use of adjuvant radiotherapy. However, due to confounding, overall survival outcomes between patients receiving surgery alone and those receiving surgery with adjuvant radiotherapy are not causally interpretable.

**Key Points**

- High-grade spinal meningiomas (SMs) have a predilection toward younger and male patients, when compared to low-grade ones.
- Adjuvant radiation does not seem to offer any benefit in terms of overall survival in the management of high-grade SMs.

**Importance of the Study**

This nationwide NCDB study on 13 184 patients offers insight as to the differences in demographics between high-grade (World Health Organization [WHO] grades 2 or 3) spinal meningiomas (SMs), in comparison to lower, WHO grade 1, SMs. In fact, patients harboring high-grade SMs were significantly younger and more commonly males. The findings also suggest no overall survival benefit associated with the use of adjuvant radiotherapy for the treatment of high-grade SMs. This ought to be interpreted with caution due to the plausible presence of unaccounted confounders.

Meningiomas are tumors that originate from arachnoid cap cells in the leptomeninges. Although spinal meningiomas (SMs) are 10 to 50 times less prevalent than their intracranial counterparts, they prevail as the most common intradural extramedullary tumor.^1^ Meningiomas, including spinal ones, are known for their female predilection, a feature that has been linked to the presence of estrogen and progesterone receptors on meningioma tumor cells.^2^ Additionally, they disproportionately affect individuals of older ages with the peak incidence at the seventh decade of life.^1^

Based on tumor behavior and histopathological features, 3 grades of SMs are recognized by the World Health Organization (WHO) classification of tumors of the central nervous system; grades 1 to 3 representing benign, atypical, and malignant tumors, respectively.^3,4^

Up to 95% of SMs are classified as benign WHO grade 1 tumors.^1^ These tumors are typically slow-growing and exhibit low proliferation (MIB-1) indices.^5^ Since higher-grade SMs (WHO grades 2 and 3) are much rarer, there is a paucity of available literature.^6^ In a recent systematic review and pooled analysis, only 267 cases of higher-grade SMs were reported among a total of 5641 tumors (5%), with WHO grade 2 SMs (*n* = 243) being 10 times as frequent as grade 3 SMs (*n* = 24).^1^ In contrast to their benign counterparts, these high-grade tumors have distinct epidemiological features,^1^ and are associated with particularly dismal prognosis.^7–9^ In fact, a recent meta-analysis found WHO grade 2 and 3 SMs to be associated with a significant 10-fold increase in the odds of recurrence, compared to WHO grade 1 SMs.^10^

Currently, limited information is available regarding the optimal management of these aggressive tumors.^6^ While the primary treatment is radical, Simpson grades of 1 or 2, tumor resection, the role of adjuvant treatments is poorly understood (Figure 1). Consequently, recommendations regarding adjuvant treatment modalities, such as radiotherapy or chemotherapy are lacking. Currently, the use of these treatment modalities is largely grounded on non-empirical evidence, and insights extrapolated from research on intracranial meningiomas.^11^

**Figure 1.**
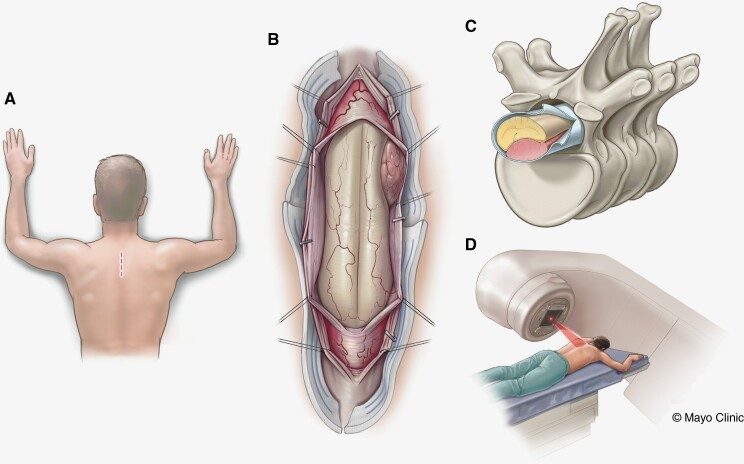
Illustrations showing the skin incision markings (A), the intradural operative view of a ventral spinal meningioma (B), the anatomical location of the tumor in relation to the spinal elements (C), and the patient receiving adjuvant radiotherapy (D).

The aim of this study was to review the nationwide experience on the treatment of high-grade SMs, investigate the associated demographics, and compare the overall survival outcomes of surgery with adjuvant radiotherapy to surgery alone in propensity score-matched cohorts.

## Methods

### Database and Ethics

The National Cancer Database (NCDB) was queried for patients diagnosed with SM between 2004 and 2017. The NCDB is one of the largest cancer registries in the US. ICD-O-3 histological codes designating meningioma (9530–9535 and 9537–9539) and ICD-O-3 topographical codes for spinal meninges (C70.1), spinal cord (C72.0), and cauda equina (C72.1) were utilized to identify relevant cases. This research is performed in accordance with all ethical guidelines. Approval was exempted by the Mayo Institutional review board as the NCDB database only contains de-identified data. Informed consent is not applicable as the data was sourced from a registry.

### Variables and Primary Outcome

Baseline characteristics including patient-specific data points, such as age, sex, race, ethnicity, Charlson–Deyo comorbidity index, income quartile, insurance status, treating facility type, and distance to facility were all extracted from the NCDB. Moreover, for tumor characteristics, WHO grade, histological subtype, tumor size (mm), and metastatic status were recorded. WHO grade 1 SMs were defined as low-grade, while WHO grades 2 and 3 were defined as high-grade. Treatment-specific data were also collected, and included treatment type, extent of tumor resection, days to start and duration of radiotherapy, number of fractions, and radiation doses (cGy). Overall survival was the primary outcome of the study.

Patients with missing data for the primary outcome were excluded from the analysis. In addressing missing data for other variables, imputation was not used. Instead, we deliberately retained “missing” as a distinct category and integrated it into the analysis, when necessary. By treating missing values as a separate entity, we sought to ensure transparency in our reporting and acknowledge the inherent uncertainty associated with unobserved data points. Nonetheless, the data completeness rate was high and amounted to 93.16%.

### Patient Cohort

In the first part of the study detailing demographic differences between low- and high-grade SMs, all patients with a diagnosis of SM retrieved from the NCDB were included. In the second part of the study, only patients with WHO grade 2 and 3 SMs were included. Moreover, patients receiving any other treatment than surgery with or without adjuvant radiotherapy were excluded.

### Statistics

The Shapiro–Wilk test was used to evaluate the normality of the data. As all continuous data significantly deviated from a normal distribution pattern (Shapiro–Wilk test *P* value < .05), it is presented as a median with interquartile range (IQR) and categorical data as numbers (proportion). The Mann–Whitney *U*, Chi squared, or Fisher’s exact test were used for between-group comparisons, as appropriate. Propensity score was employed to create balanced comparison groups based on the following covariates: age, sex, race, extent of tumor resection, Charlson–Deyo comorbidity index score, histological subtype, WHO grade, and presence of metastasis at diagnosis. Despite incomplete data for histological subtypes and extent of resection, these variables were still incorporated into the model. This inclusion aimed to adjust for these covariates to the greatest extent possible, ensuring a more comprehensive analysis. For that the “MatchIt” package in R was utilized to perform a 1:1 matching, leveraging the “optimal” matching method. After matching, standardized mean difference comparison and Love plots were performed to ensure a balanced distribution of the covariates. Kaplan–Meier survival analysis was conducted on both pre- and postmatched cohorts to determine the overall survival over follow-up time. Statistical significance was set at *P* < .05 All analyses were conducted using the statistical software program R version 4.0.5.

## Results

### Demographic and Baseline Characteristics

#### Low- versus high-grade spinal meningiomas

**Figure 2.**
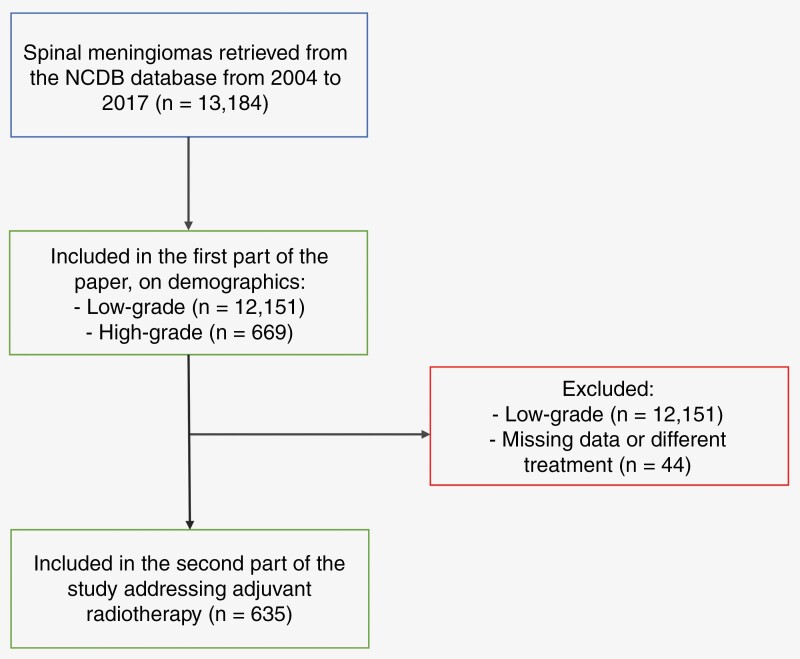
Inclusion flowchart.

A total of 13 184 patients diagnosed with SMs were identified using the NCDB database (Figure 2). The majority of the patients (*n* = 12 151; 95%) were diagnosed with low-grade (WHO grade 1) SMs, only a minority (*n* = 669; 5%) had high-grade (WHO grade 2 or 3) SMs. The ratio of WHO grade 2 to 3 SMs was 4:1. Patients with high-grade SMs presented at a younger median age (57 years (44–68) versus 65 years (54–75); *P* < .001). Males were significantly overrepresented among the high-grade group, as compared to the low-grade one (33% vs 20%; *P* < .001). There were no racial differences between the 2 groups (*P* = .44). However, Hispanic ethnicity was more commonly found among patients with high-grade SMs (*P* = .024).

Furthermore, it was shown that patients with high-grade SMs were significantly more likely to receive care in an academic program as compared to patients with low-grade SMs (51% vs 44%; *P* = .003). These patients lived further from the treating facility as compared to patients with low-grade SMs (14.1 miles (6.2–37.1) vs 12.4 (5.4–31.4); *P* = .03). Also, private insurance coverage was significantly higher among patients with high-grade as compared to those with low-grade SMs (51% vs 42%; *P* < .001).

Nonetheless, the distribution of living areas (metro vs rural vs urban) or income quartiles (Q1 vs Q2 vs Q3 vs Q4) did not differ among groups (*P* = .82 and *P* = .68; respectively). Patients in both groups had a similar distribution of Charlson–Deyo comorbidity index scores (*P* = .35).

Finally, patients with high-grade SMs exhibited significantly larger tumor diameter (21.0 mm (15–30) vs 18.0 mm (13–23); *P* < .001).

#### Surgery-alone versus surgery with adjuvant radiotherapy

After excluding patients with missing data and those receiving other treatment modalities, 620 patients with high-grade SMs remained (Table 2). Among these, 517 (83%) underwent surgery alone while 108 (17%) received both surgery and adjuvant radiotherapy. Patients in the surgery and radiotherapy group were significantly younger as compared to those in the surgery-alone group (median: 53.0 (40.0–63.0) vs 59.0 (46.0–68.0); *P* = .002). Although males were in minority, they were significantly more represented in the surgery and radiotherapy group as compared to the surgery-alone group (46% vs 30%; *P* = .001). Significant differences were observed in the distribution of race among groups (*P* = .007). Specifically, 76%, 19%, and 3% of the surgery and radiotherapy cohort were White, Black, and Asian, respectively, as compared to 87%, 8%, and 3% in the surgery-alone group.

There were no differences in comorbidity levels between the groups as determined by the Charlson–Deyo comorbidity index (*P* = .83). In both surgery-alone and surgery and radiotherapy groups, most patients had a Charlson–Deyo score of 0 (74% and 77%), followed by 1 (15% and 13%), 2 (8% and 8%), and ≥3 (4% and 2%). The tumor’s largest diameter was significantly greater in the surgery and radiotherapy group (median: 25.0 mm (18.3–43.8), compared to the surgery-alone group (median: 20.0 mm [15.0–29.0], *P* = .001).

Moreover, a comparison of the WHO grades (2 and 3) between patients undergoing both surgery and radiotherapy versus those undergoing surgery-alone revealed no significant difference (*P* = .42). In total, there were 520 (84%) and 115 (18%) patients harboring high-grade SM in WHO grades 2 and 3, respectively. On further histological classifications, the distribution between the 2 groups significantly differed (*P* < .001), with the rhabdoid/papillary subtype being more commonly offered surgery and radiotherapy.

Four patients (1.9%) had metastases at the time of diagnosis in the surgery and radiotherapy group as compared to 2 (0.8%) in the surgery-only group (*P* = .28).

After propensity-score 1:1 matching, a balanced distribution (*P* ≥ .05) across all baseline patient characteristics was achieved between the groups (Table 1 and Supplementary Figure A).

**Table 1.** Differences in Baseline Characteristics Between Low (WHO Grade 1) and High-Grade (WHO Grades 2 or 3) Spinal Meningiomas (SMs)

Variable	Overall, *N* = 13 184	Low-Grade SMs, *N* = 12 515	High-Grade SMs, *N* = 669	*P* value
Age	65.0 (54.0, 74.0)	65.0 (54.0, 75.0)	57.0 (44.0, 68.0)	**<.001**
Male	2749 (21%)	2529 (20%)	220 (33%)	**<.001**
Race				.44
Asian	382 (2.9%)	363 (2.9%)	19 (2.9%)	
Black	1091 (8.4%)	1025 (8.3%)	66 (10%)	
Other	242 (1.9%)	230 (1.9%)	12 (1.8%)	
White	11 248 (87%)	10 695 (87%)	553 (85%)	
Hispanic ethnicity	685 (5.5%)	638 (5.4%)	47 (7.5%)	**.024**
Treating facility type				**.003**
Community cancer program	520 (4.3%)	499 (4.3%)	21 (3.9%)	
Comprehensive community cancer program	4500 (37%)	4334 (37%)	166 (31%)	
Academic program	5361 (44%)	5083 (44%)	278 (51%)	
Integrated network program	1813 (15%)	1738 (15%)	75 (14%)	
Insurance status				**<.001**
Not insured	322 (2.5%)	300 (2.4%)	22 (3.3%)	
Private	5530 (43%)	5198 (42%)	332 (51%)	
Medicaid	731 (5.6%)	673 (5.5%)	58 (8.8%)	
Medicare	6236 (48%)	6002 (49%)	234 (36%)	
Other	153 (1.2%)	142 (1.2%)	11 (1.7%)	
Income quartiles				.68
Q1	1887 (16%)	1781 (16%)	106 (17%)	
Q2	2490 (21%)	2366 (21%)	124 (20%)	
Q3	2909 (24%)	2768 (24%)	141 (23%)	
Q4	4639 (39%)	4403 (39%)	236 (39%)	
Living area				.82
Metro	10 790 (85%)	10 255 (85%)	535 (84%)	
Rural	234 (1.8%)	223 (1.8%)	11 (1.7%)	
Urban	1698 (13%)	1608 (13%)	90 (14%)	
Missing)	462	429	33	
Distance from residence and hospital (miles)	12.5 (5.4, 31.8)	12.4 (5.4, 31.4)	14.1 (6.2, 37.1)	**.003**
Charlson–Deyo comorbidity index				.35
0	9629 (73%)	9131 (73%)	498 (74%)	
1	2230 (17%)	2132 (17%)	98 (15%)	
2	898 (6.8%)	846 (6.8%)	52 (7.8%)	
≥3	427 (3.2%)	406 (3.2%)	21 (3.1%)	
Tumor largest diameter (mm)	18.0 (13.0, 23.0)	18.0 (13.0, 23.0)	21.0 (15.0, 30.0)	**<.001**

Bold indicates significant *P*-values (<0.05).

**Table 2.** Baseline Characteristics of High-Grade Spinal Meningiomas Treated With Either Surgery-Alone or Surgery With Adjuvant Radiotherapy

Variable	Prematching			Postmatching		
	Surgery, *N* = 517	Surgery + radiotherapy, *N* = 108	*P* value	Surgery, *N* = 95	Surgery + radiotherapy, *N* = 95	*P* value
Age	59.0 (46.0, 68.0)	53.0 (40.0, 63.0)	**.002**	51.0 (36.0, 62.0)	52.0 (38.0, 63.0)	>.99
Male	156 (30%)	50 (46%)	**.001**	42 (44%)	46 (48%)	.56
Race			**.007**			.50
White	452 (87%)	82 (76%)		78 (82%)	72 (76%)	
Black	41 (7.9%)	20 (19%)		14 (15%)	18 (19%)	
Asian	16 (3.1%)	3 (2.8%)		3 (3.2%)	3 (3.2%)	
Other	8 (1.5%)	3 (2.8%)		0 (0%)	2 (2.1%)	
Charlson–Deyo comorbidity index			.83			>.99
0	380 (74%)	83 (77%)		71 (75%)	72 (76%)	
1	77 (15%)	14 (13%)		15 (16%)	14 (15%)	
2	41 (7.9%)	9 (8.3%)		8 (8.4%)	8 (8.4%)	
≥3	19 (3.7%)	2 (1.9%)		1 (1.1%)	1 (1.1%)	
Tumor largest diameter (mm)	20.0 (15.0, 29.0)	25.0 (18.3, 43.8)	**.001**	21.5 (15.3, 30.8)	25.0 (18.0, 47.8)	.057
Missing	217	38		41	37	
WHO grade			.42			>.99
2	433 (84%)	87 (81%)		74 (78%)	74 (78%)	
3	84 (16%)	21 (19%)		21 (22%)	21 (22%)	
Histology			**<.001**			.99
Atypical	176 (34%)	35 (32%)		30 (32%)	29 (31%)	
Anaplastic	72 (14%)	14 (13%)		15 (16%)	14 (15%)	
Clear cell/chordoid	73 (14%)	13 (12%)		9 (9.5%)	11 (12%)	
Rhabdoid/papillary	4 (0.8%)	6 (5.6%)		4 (4.2%)	6 (6.3%)	
Unspecified	192 (36.7%)	40 (36.9%)		37 (39.1%)	35 (37.1%)	
Metastases at diagnosis	4 (0.8%)	2 (1.9%)	.28	1 (1.1%)	2 (2.1%)	>.99
Extent of resection			**<.001**			.94
Gross total	52 (10%)	9 (8.3%)		5 (5.3%)	6 (6.3%)	
Subtotal	18 (3.5%)	14 (13%)		13 (14%)	12 (13%)	
Unspecified	447 (86%)	85 (79%)		77 (81%)	77 (81%)	

Bold indicates significant *P*-values (<0.05).

### Treatment Specifications

Extent of resection was unspecified in over 80% of patients. The median time from diagnosis to the start of radiotherapy was 64.0 days (43.0–109.0), while the median duration of the radiotherapy itself, from start to finish, was 39.0 days (36.0–43.0). The majority of patients (93%) received external beam radiation, while the rest (7%) received stereotactic radiosurgery. The median number of radiation fractions was 28.0 (25.0–29.0). The median total dose administered was 5040 cGy (4500–5400) (Table 3).

**Table 3.** Radiotherapy Specifications in the Matched Cohort

Variable	Postmatching Cohort *N* = 95
From diagnosis to start of radiation (days)	64.0 (43.0, 109.0)
Missing	8
Days from start to end of radiation therapy	39.0 (36.0, 43.0)
Missing	10
Radiation type	
External beam	88 (93%)
SRS	7 (7.4%)
Number of fractions	28.0 (25.0, 29.0)
Missing	26
Total dose (cGy)	5040.0 (4500.0, 5400.0)
Missing	14

### Survival Analysis

**Figure 3.**
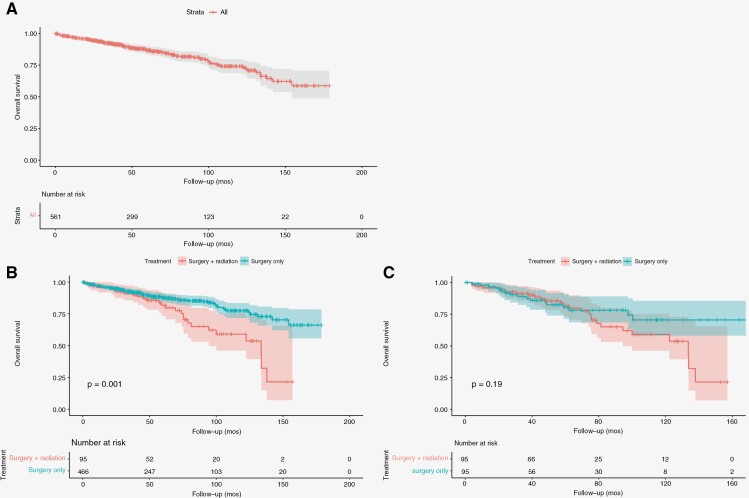
Kaplan–Meier survival curves showing overall survival over time (months), for the high-grade SM cohort prior to matching (A), stratified by treatment type and prior to matching (B), and stratified by treatment and after propensity score matching (C).

Patients were followed for an average of 60.5 months (36–89), with no differences in follow-up length between groups both prior to (*P* = .57) and after propensity score matching (*P* = .22). In the matched cohort, the 30-day and 90-day mortality rates were 0% regardless of the treatment group. On Kaplan–Meier survival analysis, a 5-year and 10-year overall survival rate of around 85% and 75% were seen for high-grade SM treated with either surgery or surgery with adjuvant radiotherapy, respectively (Figure 3). Prior to matching, survival analysis revealed significantly worse overall survival outcomes in patients undergoing surgery with adjuvant radiotherapy as compared to surgery-alone (*P* = .001). However, after propensity-score matching, the difference in overall survival was not retained (*P* = .19).

## Discussion

This study, to the best of our knowledge, describes the largest cohort of high-grade SMs to date. In this material, high-grade (WHO grade 2 or 3) SMs constituted 5.1% of all SMs, which corroborates previous estimates.^1^ Major demographic differences were noted between patients with high-grade and those with low-grade SMs. Patients with high-grade SMs were, to higher extents, younger, and males. These patients traveled further distances to receive care and were more likely to be admitted in an academic hospital. Although some studies have previously addressed the survival of patients with high-grade SMs,^12,13^ our study is the first, to our knowledge, to compare the overall survival in adjusted and propensity score-matched cohorts of patients undergoing either surgery alone or surgery and adjuvant radiotherapy. Our postmatching results indicate no difference in overall survival in patients with high-grade SMs treated with surgery and adjuvant radiotherapy as compared to surgery-alone.

This study revealed baseline demographic differences between high- and low-grade SMs. While low-grade SMs are well known to exhibit a strong female predilection, sex was almost evenly distributed in patients with high-grade SMs, with 46% of patients being male. In comparison, only 30% of patients with low-grade SMs were males, with the difference being significant (*P* = .001). Patients with high-grade SMs had a younger median age compared to low-grade patients (*P* = .001). In 2 pediatric case series involving a pooled total of 24 tumors, 8 tumors (33%) were classified as WHO grade 2, indicating a marked overrepresentation of high-grade tumors in this cohort.^14,15^ It is worth noting that this overrepresentation persisted, even when patients with neurofibromatosis or multiple meningiomas were excluded from the study.^14^ In contrast, a pooled proportion of higher-grade SMs of 3.2% was found among 2 studies on the elderly population.^16,17^ Also, a significantly higher proportion of high-grade SMs was detected among younger patients in 2 studies comparing younger (<50 years) and older SM patients (≥50 years).^18,19^ In the pooled younger cohort, 5.7% of patients had a high-grade SM versus 0.8% of older patients. In keeping with that, numerous studies on high-grade SMs have consistently reported relatively lower mean ages as compared to the general population of patients with meningioma.^20^ For instance, a study on 12 patients with WHO grade 2 clear cell SMs noted an average age of 28.8 years.^21^ Three additional studies involving mixed higher grades (WHO grades 2 and 3) on 16, 19, and 25 patients reported mean ages of 52.8,^22^ 37.8,^20^ and 46.6 years,^23^ respectively. One final study on 6 patients with WHO grade 3 SMs revealed an average age of 29 years among patients.^8^

Furthermore, our study highlighted an overrepresentation of males among high-grade SMs as compared to low-grade ones. These findings are in accordance with previous literature revealing a relatively high proportion of males in high-grade SM cohorts. The proportion of male patients in 3 studies on high-grade SMs ranged from 42% to 50%,^8,21,22^ which is considerably higher than the 28% proportion typically seen in the general population of patients with SM. High-grade SMs are more often found in pediatric reports and among younger patients. Overall, a striking overrepresentation of high-grade SM was evident among male and younger patients in this study.

In previous studies, radiotherapy has been offered for a wide variety of indications including high-grade tumors, recurring tumors, or those subtotally resected. Some have even indicated the use of radiation therapy alone, especially in patients with neurofibromatosis or multiple SMs.^10^ While the literature indicates an increase in the use of adjuvant radiotherapy for the treatment of SMs during recent years,^13^ this gain in popularity could not be correlated to any novel evidence on the topic. Instead, this increase likely stems from evidence originating from research on intracranial meningiomas, where studies have consistently shown positive results. In fact, a recent meta-analysis gathering the evidence from 13 different retrospective studies on 1113 patients with high-grade intracranial meningiomas, revealed significant benefits in terms of overall and progression-free survival, and tumor control, associated with the use of adjuvant radiotherapy.^11^ Meanwhile, as opposed to cranial ones, evidence supporting the use of adjuvant radiotherapy for high-grade SMs remains nonexistent. The difference in treatment response between these 2 counterparts may be related to different underlying biological pathways and tumor behaviors.^24,25^

On par with the initial survival analysis conducted on the unmatched cohorts in this study, other studies have shown significantly worse outcomes associated with the use of adjuvant radiotherapy in patients undergoing surgery for high-grade SMs.^13^ The result from this prematching analysis stems from an inherent selection bias, whereby patients receiving both surgery and adjuvant RT were more likely to be those with more advanced disease processes, larger tumors, and those receiving subtotal tumor resections. Oppositely, those receiving surgery alone were more likely to have localized as well less advanced tumors, and undergo gross total resection. In an attempt to calibrate the cohorts in that specific aspect, matching was performed and revealed no significant difference in overall survival between the groups. Although adjustment using propensity score matching resulted in a shift of direction toward nonsignificant differences, it was impossible to identify the isolated effect of adjuvant radiotherapy on survival outcomes, based on this study. This is mainly due to lack of granularity and numerous gaps in the database. These results highlight the need for future prospective studies to determine the true role of adjuvant radiotherapy in the treatment of high-grade SMs.

### Limitations

This study has several limitations, mainly inherent to database studies in general. First, several patients with missing data had to be excluded. It is of note that a previous study has suggested a significant bias toward worse outcomes in patients with missing data in the NCDB.^26^ The absence of data pertaining to the extent of tumor resection, histological subtypes, recurrence status, and other relevant variables hindered the possibility of conducting a comprehensive subgroup-level analysis. Although robust for studying long-term outcomes, the NCDB has flaws, in terms of the lack of both baseline patient data such as detailed comorbidity data, as well as outcome data, including surgical, medical complications, and tumor recurrence. Patient-reported outcomes and health-related quality-of-life measures are not included in the NCDB. Particularly, the lack of baseline data limits our ability to adjust for unmeasured covariates that can have an influence on study outcomes. Noting the difference in unmatched survival between patients receiving surgery alone and those receiving surgery and adjuvant radiotherapy raises concerns of possible confounding bias and overall survival outcomes are not causally interpretable.

## Conclusions

This study reports on the largest cohort of high-grade SMs to date. A marked overrepresentation of younger and male patients was found among high-grade SMs. Adjuvant radiotherapy did not significantly affect the overall survival. However, the registry data was not granular enough to allow causal interpretation of the findings.

## Supplementary Material

vdae018_suppl_Supplementary_Figure

## Data Availability

The data supporting this research may be provided upon reasonable request.
