# Supplementary figures and images for: Analysis of demographics and the impact of adjuvant radiotherapy on a nationwide cohort of patients with high-grade spinal meningiomas
Source: Neurooncol Adv. 2024 Feb 5;6(1):vdae018. doi: 10.1093/noajnl/vdae018 (PMC10896623; doi:10.1093/noajnl/vdae018)

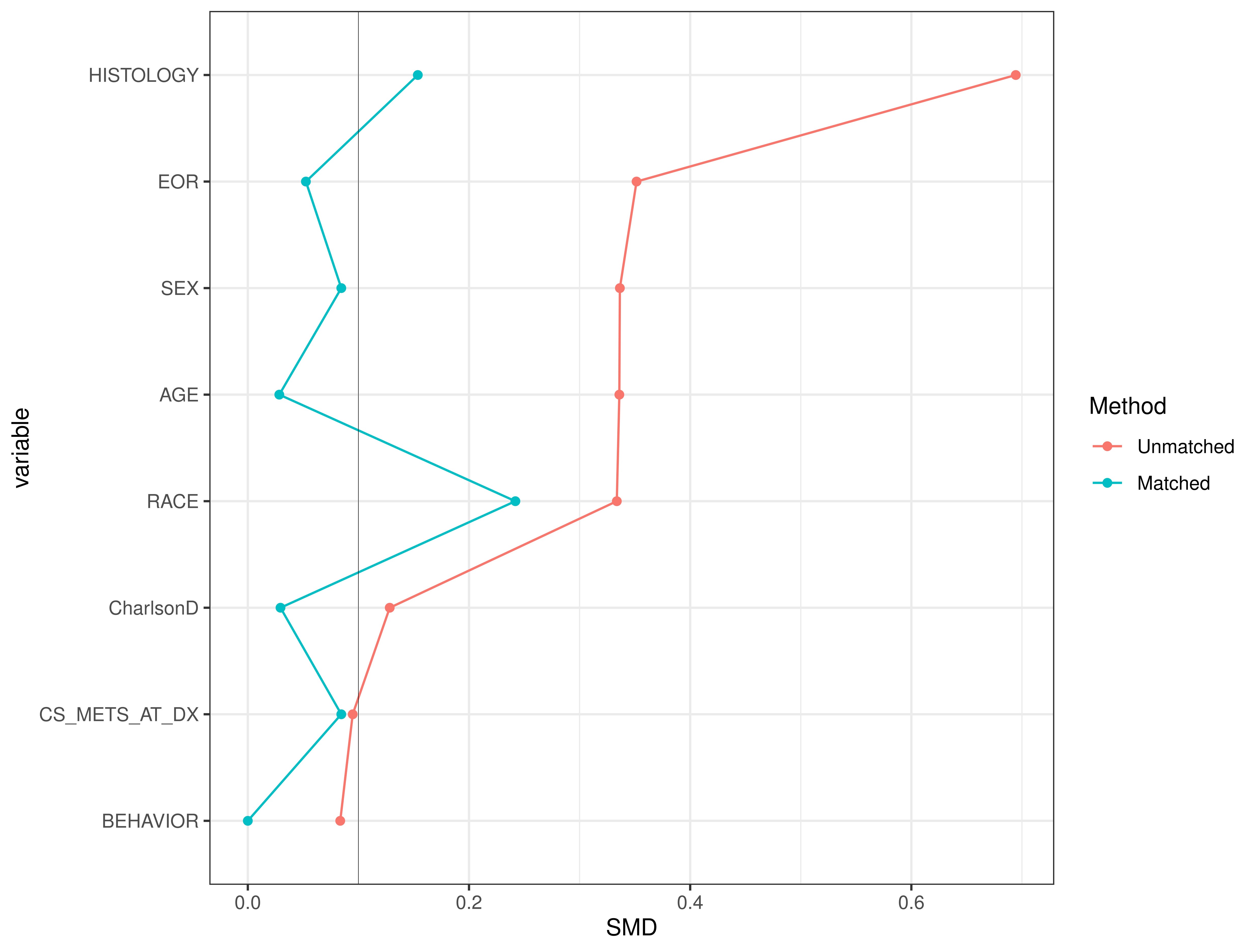

Supplement: vdae018_suppl_Supplementary_Figure [file vdae018_suppl_supplementary_figure.jpeg]
